# Supplementary material for: The fnr‐like mutants confer isoxaben tolerance by initiating mitochondrial retrograde signalling
Source: Plant Biotechnol J. 2024 Jun 27;22(11):3000–11. doi: 10.1111/pbi.14421 (PMC11500984; doi:10.1111/pbi.14421)
Supplement: Supplementary file 1 — Figure S1 fnrl‐1 complementation, T‐DNA insertion sites and FNRL expression in fnrl mutants. Figure S2 Isoxaben dose–response and cross‐tolerance to other CBIs in fnrl mutants. Figure S3 Lambda scans of FNRL:GFP transiently expressed in Nicotiana benthamiana leaves. Figure S4 Chloroplast retrograde signalling pathways are not active in the fnrl mutants. Figure S5 Phylogenetic analysis of FNRL homologues in plant lineages. [file PBI-22-3000-s002.docx]

A.


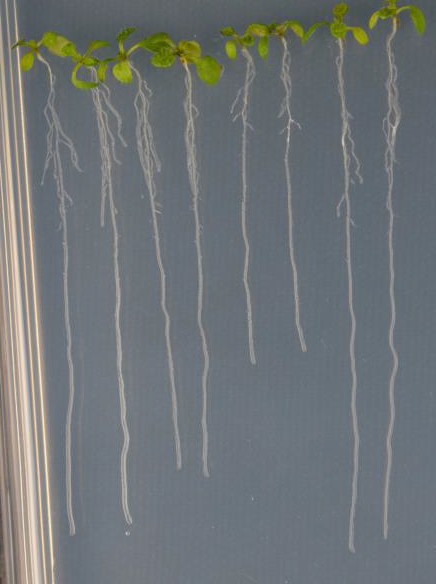

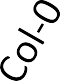

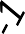

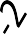

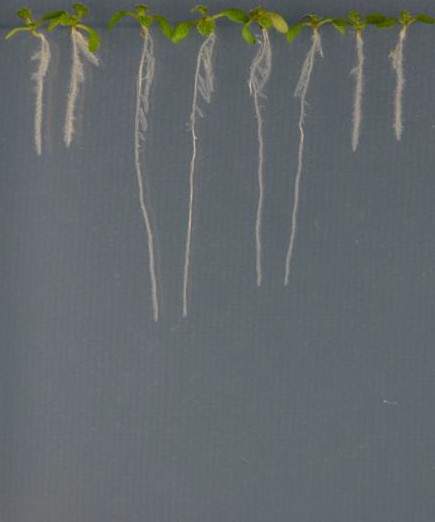

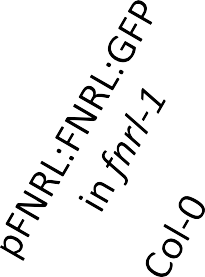

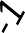

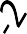

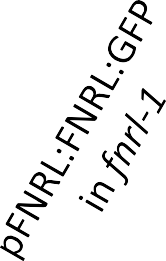


B.

*fnrl-1 fnrl-2*

*FNRL*


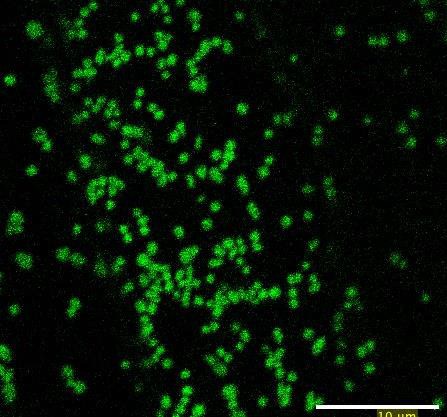
D.

Mock

2.5nM

ISX


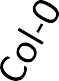
C.


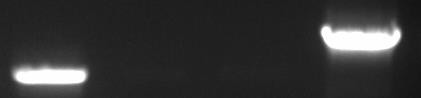

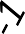

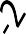


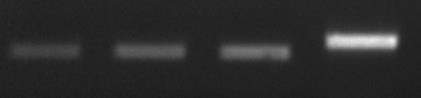
*FNRL UBQ10*

# Supplemental Figure 1: *fnrl-1* complementation, T-DNA insertion sites, and *FNRL* expression in *fnrl*

**mutants**

1. Root length phenotype of indicated genotypes grown on Mock and 2.5 nM isoxaben (ISX) media for

10 days.

1. T-DNA insertion sites of *fnrl-1* and *fnrl-2* alleles in the *FNRL* gene.
2. Expression of *FNRL* in *fnrl-1* and *fnrl-2* mutants compared to the wild type. RNA was extracted from the roots of 7-day-old seedlings. Reverse transcription was performed on 1 µg of RNA, followed by standard PCR using full-length *FNRL* primers spanning from the ATG start codon to the stop codon. UBQ10 primers were used as a control, and genomic DNA (gDNA) was used as a control for gDNA contamination.
3. FNRL:GFP localisation in 5 days old Arabidopsis roots. Scale bar = 10um


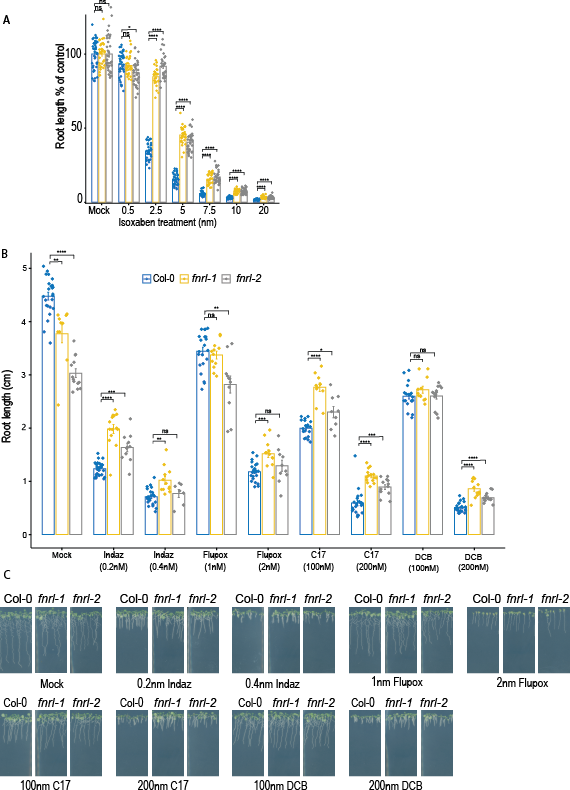


**Supplemental Figure 2**: **Isoxaben dose response and cross tolerance to other CBIs in *fnrl* mutants.**

- 1. Primary root length as a percentage of the control under a range of isoxaben concentrations.
  2. Primary root length of ten-day-old seedlings of Col-0, *fnrl-1*, and *fnrl-2* under different CBIs and mock treatment. Statistical significance is indicated by asterisks (*, P < 0.05; **, P < 0.01; ***, P < 0.001; and ****, P < 0.0001) based on Student’s t-test.
  3. Representative images of ten-day-old seedlings of Col-0, *fnrl-1*, and *fnrl-2* under different CBIs and mock treatment.


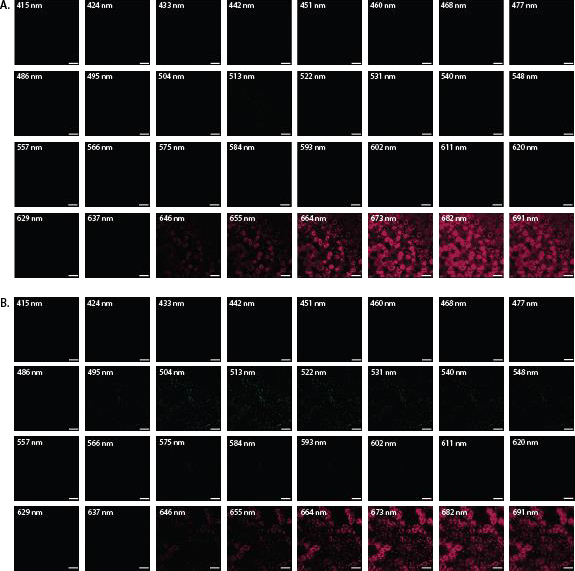


**Supplemental Figure 3:** Lambda scans of FNRL:GFP transiently expressed in *Nicotiana benthamiana* leaves.

(A) Control and (B) FNRL:GFP infiltrated leaf. Excitation was performed with a 488 nm laser. The GFP signal between 500 and 550 nm is only detected in FNRL:GFP-infiltrated leaves, while chlorophyll autofluorescence is detected in both control and FNRL:GFP samples. Scale bars = 50 µm.

A.

Col-0

Mock

*gun1*

Col-0

Lin

*gun1*


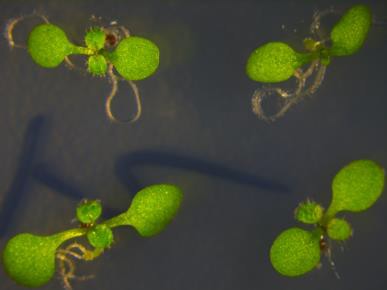

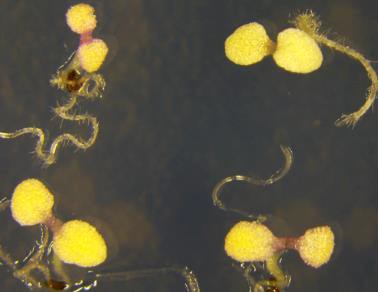


*fnrl-1 fnrl-2 fnrl-1 fnrl-2*


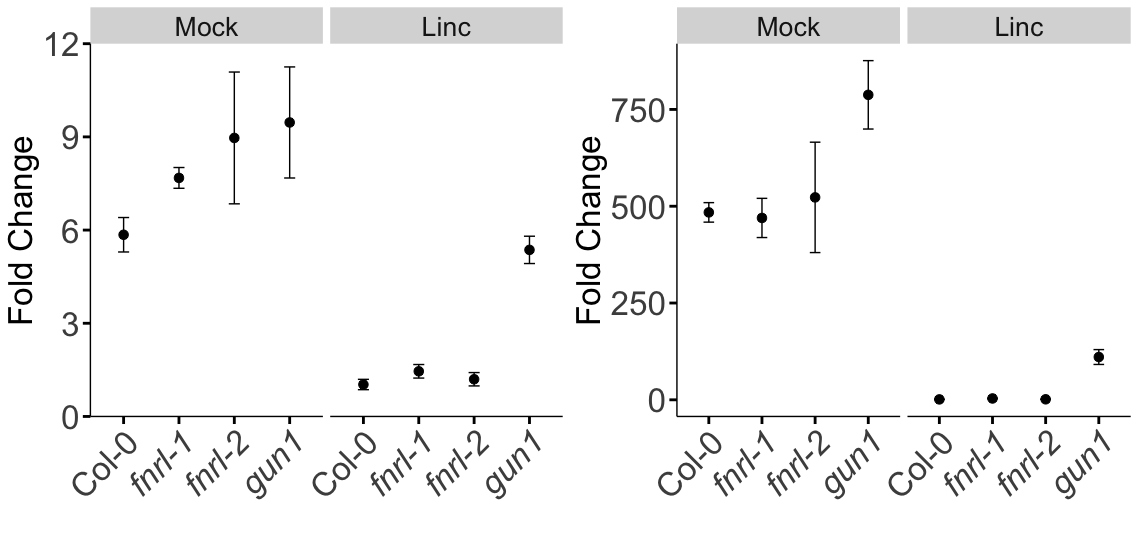


B.

CP12

LHCB1.1

ns ns

ns

ns

ns

ns

**

ns

ns

**

ns ns

# Supplemental Figure 4: chloroplast retrograde signalling pathways are not active in the *fnrl* mutants.

1. Representative images of Arabidopsis wild type (Col-0), *gun1*, *fnrl-1*, and *fnrl-2* seedlings grown in the

absence or presence of 550 μM lincomycin (Linc).

1. Gene expression levels of chloroplast retrograde signalling marker genes measured by qRT-PCR in seven-day-

old seedlings treated with 550 μM lincomycin or mock. The results are shown for three biological replicates. Mean ± SD is presented. Statistical significance is indicated by asterisks (ns, non-significant; *, P < 0.05; and **, P < 0.01) based on Student’s t-test.

#
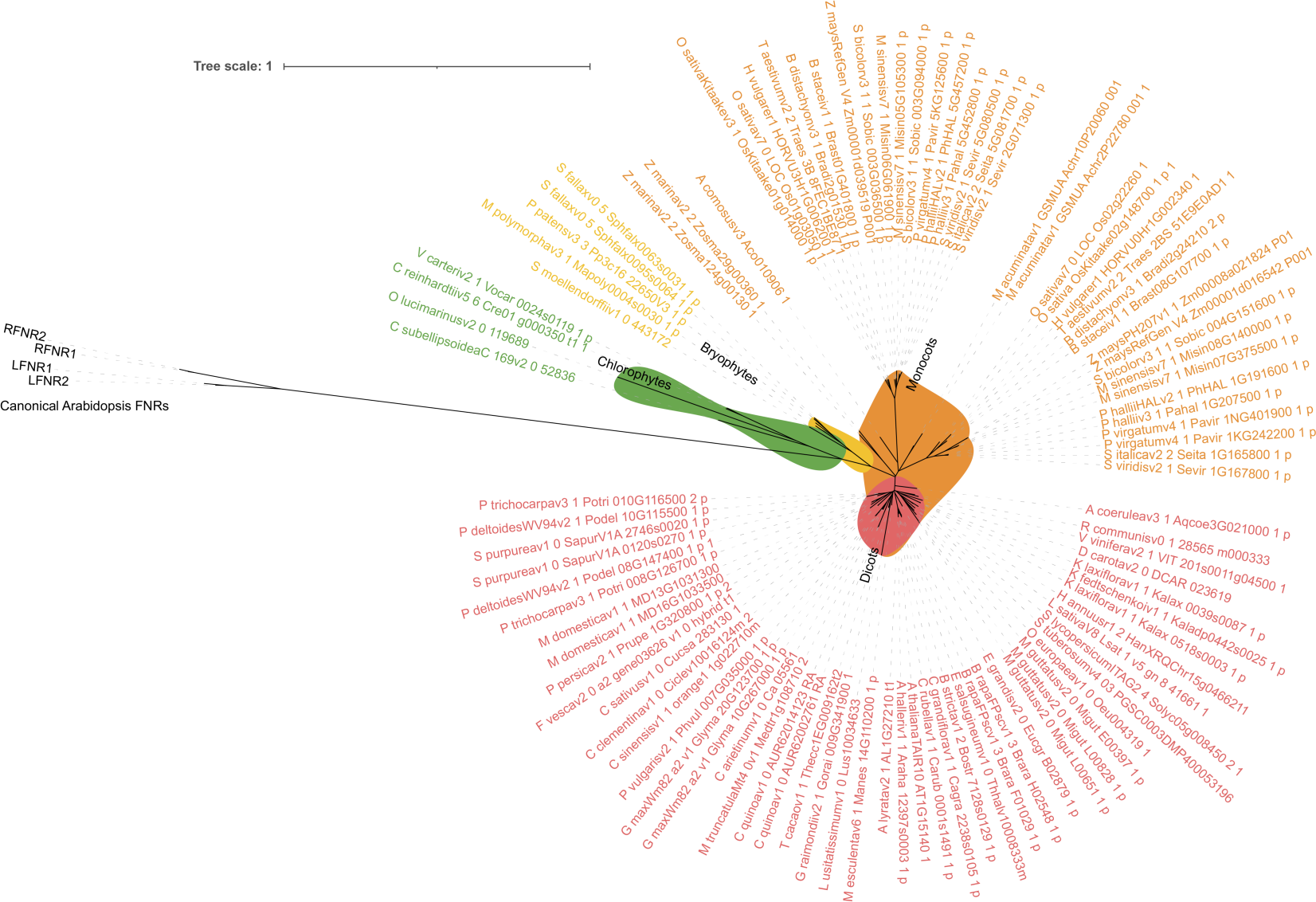
Supplemental Figure 5: Phylogenetic analysis of FNRL homologues in plant lineages.

Unrooted phylogenetic tree of FNRL homologues and FNR genes across diverse plant lineages. We conducted phylogenetic analyses using the FNRL amino acid sequence, searching for homologues in Phytozome across 62 Viridiplantae species. Sequences, including canonical FNRs from Arabidopsis, underwent alignment and tree construction using NGphylogeny. The resulting tree was visualized in iTOL and minor aesthetic adjustments were made in Adobe Illustrator.
