# Supplementary material for: The fnr‐like mutants confer isoxaben tolerance by initiating mitochondrial retrograde signalling
Source: Plant Biotechnol J. 2024 Jun 27;22(11):3000–11. doi: 10.1111/pbi.14421 (PMC11500984; doi:10.1111/pbi.14421)
Supplement: Supplementary file 2 — Table S1 List of FNRL homologues identified in diverse plant lineages and their similarity scores. Table S2 Primers used in this study. [file PBI-22-3000-s001.docx]

Table S2: List of FNRL homologues identified in diverse plant lineages and their similarity scores.

| **Organism** | **Gene Name** | **Phytozome Similarity Score** | **Protein**  **sequence Similarity** |
| --- | --- | --- | --- |
| Arabidopsis thaliana | AT1G15140.1 | 610 | 100 |
| Arabidopsis halleri | Araha.12397s0003.1 | 573 | 99 |
| Arabidopsis lyrata | AL1G27210.t1 | 572 | 99 |
| Coreopsis grandiflora | Cagra.2238s0105.1 | 564 | 98.6 |
| Capsella rubella | Carub.0001s1491.1 | 564 | 98.6 |
| Boechera stricta | Bostr.7128s0129.1 | 554 | 97.3 |
| Eutrema salsugineum | Thhalv10008333m | 525 | 95.6 |
| Brassica rapa | Brara.H02548.1 | 538 | 95.3 |
| Brassica rapa | Brara.F01029.1 | 489 | 94.5 |
| Cicer arietinum | Ca_05561 | 409 | 89.1 |
| Chenopodium quinoa | AUR62002761-RA | 367 | 89.1 |
| Sarracenia purpurea | SapurV1A.2746s0020.1 | 403 | 88.9 |
| Gossypium raimondii | Gorai.009G341900.1 | 455 | 88.2 |
| Eucalyptus grandis | Eucgr.B02879.1 | 445 | 88 |
| Theobroma cacao | Thecc1EG009162t2 | 426 | 87.8 |
| Populus trichocarpa | Potri.008G126700.1 | 418 | 86.9 |
| Populus deltoides | Podel.08G147400.1 | 419 | 86.9 |
| Prunus persica | Prupe.1G320800.1 | 430 | 86.3 |
| Ananas comosus | Aco010906.1 | 313 | 86 |
| Erythranthe guttata | Migut.E00397.1 | 385 | 86 |
| Cucumis sativus | Cucsa.283130.1 | 384 | 85.9 |
| Citrus clementina | Ciclev10016124m | 423 | 85.8 |
| Lactuca sativa | Lsat_1_v5_gn_8_41661 | 405 | 85.3 |
| Myrica esculenta | Manes.14G110200.1 | 433 | 85.2 |
| Malus domestica | MD13G1031300 | 424 | 85.1 |
| Citrus sinensis | orange1.1g022710m | 417 | 85.1 |
| Chenopodium quinoa | AUR62014123-RA | 386 | 85 |
| Erythranthe guttata | Migut.L00828.1 | 390 | 85 |
| Phaseolus vulgaris | Phvul.007G035000.1 | 427 | 84.8 |
| Malus domestica | MD16G1033500 | 427 | 84.7 |
| Erythranthe guttata | Migut.L00651.1 | 373 | 84.7 |
| Ricinus communis | 28565.m000333 | 414 | 84.6 |
| Solanum lycopersicum | Solyc05g008450.2.1 | 419 | 84.6 |
| Fragaria vesca | gene03626-v1.0-hybrid | 421 | 84 |
| Solanum tuberosum | PGSC0003DMT4000785 | 412 | 83.9 |
| Daucus carota | DCAR_023619 | 395 | 83.8 |
| Olea europaea | Oeu004319.1 | 369 | 83.7 |
| Glycine max | Glyma.20G123700.1 | 402 | 83.6 |
| Vitus vinifera | VIT_201s0011g04500.1 | 407 | 83.5 |
| Medicago truncatula | Medtr1g108710.2 | 376 | 83.4 |
| Populus deltoides | Podel.10G115500.1 | 419 | 83.1 |
| Linum usitatissimum | Lus10034633 | 326 | 83 |
| Populus trichocarpa | Potri.010G116500.2 | 420 | 82.7 |

| Sarracenia purpurea | SapurV1A.0120s0270.1 | 378 | 82.5 |
| --- | --- | --- | --- |
| Helianthus annuus | HanXRQChr15g046621 | 404 | 82.2 |
| Glycine max | Glyma.10G267000.1 | 415 | 82 |
| Kalanchoe fedtschenkoi | Kaladp0442s0025.1 | 403 | 81.4 |
| Kalanchoe laxiflora | Kalax.0039s0087.1 | 402 | 81.4 |
| Musa acuminata | GSMUA_Achr2T22780_ | 345 | 81.2 |
| Kalanchoe laxiflora | Kalax.0518s0003.1 | 401 | 81.1 |
| Panicum virgatum | Pavir.1NG401900.3 | 334 | 80.9 |
| Oryza sativa | LOC_Os02g22260.1 | 331 | 80.6 |
| Triticum aestivum | Traes_2DS_489826DD0 | 264 | 80.1 |
| Aquilegia coerulea | Aqcoe3G021000.1 | 397 | 79.9 |
| Musa acuminata | GSMUA_Achr10T20060 | 379 | 79.8 |
| Panicum hallii | Pahal.1G207500.1 | 333 | 79.7 |
| Brachypodium distachyo | Bradi2g24210.2 | 316 | 79 |
| Brachypodium stacei | Brast08G107700.1 | 318 | 78.6 |
| Sphagnum fallax | Sphfalx0095s0064.1 | 309 | 78.6 |
| Zostera marina | Zosma124g00130.1 | 321 | 78.3 |
| Setaria italica | Seita.1G165800.1 | 332 | 77.7 |
| Setaria viridis | Sevir.1G167800.1 | 329 | 77.3 |
| Sphagnum fallax | Sphfalx0063s0031.1 | 305 | 76.4 |
| Camellia sinensis | Misin08G140000.1 | 323 | 74.9 |
| Selaginella moellendorffi | 443172 | 291 | 74.6 |
| Sorghum bicolor | Sobic.004G151600.1 | 324 | 74.6 |
| Camellia sinensis | Misin07G375500.1 | 329 | 74.2 |
| Hordeum vulgare | HORVU0Hr1G002340.1 | 332 | 73.9 |
| Physcomitrium patens | Pp3c16_22650V3.1 | 282 | 73.8 |
| Triticum aestivum | Traes_3B_8FEC1BE87.1 | 238 | 72.8 |
| Zea mays | Zm00001d039519_T00 | 247 | 72.7 |
| Marchantia polymorpha | Mapoly0004s0030.1 | 299 | 72.7 |
| Triticum aestivum | Traes_2BS_51E9E0AD1 | 316 | 72.5 |
| Zea mays | Zm00001d016542_T00 | 307 | 71.5 |
| Oryza sativa | LOC_Os01g03050.1 | 257 | 71.4 |
| Brachypodium distachyo | Bradi2g01530.1 | 246 | 71 |
| Camellia sinensis | Misin06G061900.1 | 233 | 71 |
| Camellia sinensis | Misin05G105300.1 | 217 | 70.6 |
| Panicum hallii | Pahal.5G452800.1 | 237 | 70.6 |
| Panicum virgatum | Pavir.5KG125600.1 | 234 | 70.6 |
| Setaria italica | Seita.5G081700.1 | 236 | 70.2 |
| Sorghum bicolor | Sobic.003G036500.1 | 237 | 70 |
| Setaria viridis | Sevir.5G080500.1 | 235 | 69.7 |
| Hordeum vulgare | HORVU3Hr1G006200.1 | 246 | 68 |
| Sorghum bicolor | Sobic.003G094000.1 | 244 | 68 |
| Brachypodium stacei | Brast01G401800.1 | 250 | 67.3 |
| Zea mays | Zm00008a021824_T01 | 233 | 62.4 |
| Chlamydomonas reinhar | Cre01.g000350.t1.1 | 177 | 62.3 |
| Coccomyxa subellipsoida | 52836 | 188 | 61.8 |

| Volvox carteri | Vocar.0024s0119.1 | 176 | 61.4 |
| --- | --- | --- | --- |
| Ostreococcus lucimarinus | 119689 | 122 | 55.2 |

| **Table S3: Primers used in this study.** | | | |
| --- | --- | --- | --- |
| Primer name | Sequence | | |
| qRT TCH4 LP | GATCACTTGGGGTGATGGTC |  |  |
| qRT TCH4 RP | GGGACAAGCTTCATTTGCAT |  |  |
| qRT UPOX LP | AACCGCAAGTGCCTGCATCAT |  |  |
| qRT UPOX RP | AACTGCCTCCGCCTTGGAGAA |  |  |
| qRT ANAC013 LP | TCAACCAGTGGTGGTCGTTTCA |  |  |
| qRT ANAC013 RP | GCCTCCCTGAACCTCCCATTG |  |  |
| qRT AOX1a LP | TCGCGAAACCGAAATGGTACG |  |  |
| qRT AOX1a RP | GCCGGAGCAGGAACATTCTCA |  |  |
| qRT CP12 LP | AGCCGATTAAAGCAGCACCG |  |  |
| qRT CP12 RP | GCTAAGTTCTTCAACCTCGTCCC |  |  |
| qRT LHCB1.1 LP | CGGAAAGTGAGCCAAGTTCT |  |  |
| qRT LHCB1.1 RP | TGAAAGTCTCTACCATCCACCA |  |  |
